# Organic vs. conventional: impact of cultivation treatments on the soil microbiota in the vineyard

**DOI:** 10.3389/fmicb.2023.1242267

**Published:** 2023-10-12

**Authors:** Andrea Colautti, Marcello Civilini, Marco Contin, Emilio Celotti, Lucilla Iacumin

**Affiliations:** Department of Agricultural, Food, Environmental and Animal Science, University of Udine, Udine, Italy

**Keywords:** microbiota, soil, vineyard, organic, wine, environment

## Abstract

The aim of this study was to compare the effects of two vineyard management practices on the soil and its associated microbiota. The experiments were conducted in two adjacent plots, one completely organically managed and the other conventionally managed in terms of phytosanitary treatments but fertilized with organic amendments. The chemical soil analyses were correlated to the prokaryotic and fungal communities, which were studied using the metabarcoding technique. The main difference between the two treatments was a significantly higher amount of Cu in the organic managed vineyard soil, while conventional managed soil presented higher concentration of Na and Mg and was also associated with higher pH values. Despite these differences, no significant diversities were observed on soil biodiversity and microbial composition considering alpha and beta diversity metrics. However, the percentages of some phyla analyzed individually differed significantly between the two managements. Analyzing the metabolisms of these phyla, it was discovered an increment of species correlated to soils with higher organic matter content or land not used for agricultural purposes in the organic treated soil. The findings indicate that, despite the use of copper-based phytosanitary products, there was no degradation and loss of biodiversity in the organic soil microbial population compared to conventional management with the same type of fertilization, and the observed microbial population was more similar to that of natural soils.

## 1. Introduction

Plants and soil microbiota interact in a mutualistic way, influencing each other. Plants produce root exudates that act as signal molecules to the bacteria in the rhizosphere, and each microorganism interacts with the soil microbiome, influencing plant health and productivity (Chaparro et al., 2012). Up to 10^4^ species of prokaryotes can be present in soil with high organic content, at concentrations of up to 10^10^ cells per cm^3^ (Torsvik et al., 2002). These microbial species, particularly bacteria, are the primary source of biodiversity in the soil ecosystem, where they participate in a variety of functions and balances, including nutrient cycling and plant health. However, biodiversity is linked to a number of factors, including ecosystem stability (Maherali and Klironomos, 2007; Jing et al., 2015). A stable ecosystem is distinguished by high genetic variability, which enables resistance to environmental changes (Yang et al., 2018). Several factors, many of which are related to agricultural activities, can disrupt this balance by altering the microbial community (Allison and Martiny, 2009). For example, increased nutrient availability due to fertilization can alter species composition (Zhao et al., 2020), whereas different agricultural management systems influence the carbon activity of microbial biomass (García-Orenes et al., 2010). Furthermore, the use of synthetic fungicides and pesticides used in conventional agriculture has a significant impact on the fungal and bacterial populations of the soil (Sigler and Turco, 2003; Rivera-Becerril et al., 2017). The loss of some species specialized in unique biochemical processes, such as nitrogen fixation or toxic compounds degradation, can result in nutrient loss and toxins accumulation. Fortunately, for the most important functions, the soil microbiota can compensate for this loss through functional redundancy, resulting in a stable environment with high buffering capacities (Pan et al., 2014). Organic farming is one possible strategy for reducing the impact of agricultural practices on soil microbiota, as evidenced by numerous studies that found positive effects when this management was implemented. For example, it has been reported that during the transition from conventional to organic farming, the number of plant species increases (Gabriel et al., 2006), and this recovery occurs quickly (Jonason et al., 2011). Organic farming has been shown also to have a more complex bacterial network than conventional agriculture. This is a determining factor because not only the number of present taxa, but also their interconnection, influences global characteristics (Banerjee et al., 2019). When compared to mineral (i.e., inorganic) fertilizers, the use of compost in organic farming improves soil quality by activating different microbial groups, increasing functional diversity, and increasing levels of microbial organic carbon, nitrogen, and biomass (Chaudhry et al., 2012). The greater presence of phospholipid fatty acids and phospholipid ether lipids, as well as the association of a higher biomass with regard to fungi, provide additional evidence on the efficacy of manure-based fertilization in organic farming on microbial biomass and its diversity indices (Esperschütz et al., 2007). Further genomic studies have confirmed the ability of organic farming to increase wealth, reduce uniformity and dispersion, and modify the structure of the soil microbiota thanks to organic fertilizers (Hartmann et al., 2015). Organic soil management can thus increase bacterial activity and culturable bacterial counts (Reilly et al., 2013). However, copper accumulation in the soil is a factor that must be considered in organic farming (Jez et al., 2023). In facts, especially in orchards and vineyards, there is a widespread use of copper compounds used as fungicides to combat many plant diseases (Pietrzak and McPhail, 2004). After carrying out their action, copper residues are washed away from the plant and end up accumulating in the soil, up to very high levels, which in some cases reached 1,500 mg kg^−1^ (Flores-Vèlez et al., 1996).

Several studies have shown that the use of copper-based products for an extended period of time can lead to an accumulation of this potentially toxic metal in the soil, particularly on the surface (Arias et al., 2004; Lamichhane et al., 2018), with values ranging from 100 to 3,200 mg/kg (Mirlean et al., 2007). This issue is exacerbated in vineyard cultivation, which makes extensive use of Cu-based compounds, resulting toxic to plants at high concentrations. Although some studies showed that phytotoxicity and the negative effects on the microbiota are limited (Ruyters et al., 2013), the interaction between plants and bacteria in the rhizosphere, which can increase the tolerance to copper and detoxify the soil is critical in this case (Brunetto et al., 2016). For these reasons, it is important to understand the significance of soil bacteria in sustainable and low-input cultivation systems. In fact, in addition to the need to select specific plant characteristics for organic farming (Van Bueren et al., 2002), the role of these microorganisms in maintaining soil fertility and biocontrol of pathogenic microorganisms to limit pesticides use is critical (Hacquard et al., 2017). As a result, it is becoming increasingly important to understand and monitor the microbiota present in the rhizosphere, especially given the importance of mycorrhizal colonization for plant development and health, which compensates for a lower control of pathogens with pesticides and increases the nutrients availability in these low input systems (Johansson et al., 2004). The knowledge and characterization of the soil microbiota is even more important in the wine-growing sector, as it has been demonstrated that the microbiota is a unique and characteristic biomarker for each vineyard, influencing the quality of the obtained wine both indirectly, acting on the physiology and health of the vine, and directly, acting as the main source of indigenous fermentative bacteria. Indeed, it has been reported that the microbial biodiversity associated with a specific vineyard plays a key role in plant growth, grape quality and the winemaking process (Gilbert et al., 2014; Belda et al., 2020). Also, the association between the microbial metabolic profiles of wine based on grape origin, underlines the importance of fungal community of vineyard soil in wine characterization (Liu et al., 2020).

The aim of this study is to focus on the effect of the phytosanitary treatments on the soil microbiota of organically vs. conventional managed vineyards, in particular paying attention to the possible effects of copper, the main component of organic phytosanitary treatments, to verify any positive or negative effects on vineyard soil microbial biodiversity. As far as fertilization is concerning, only few differences are applied. In fact, the fertilization of this conventional management was made up of only organic manures, thus making soil organic inputs very similar to the biological vineyards, reducing to zero level the chemicals. The chemical composition of the soil was also analyzed, to verify the possible accumulation of compounds such as Cu, and to evaluate their contribution in shaping the prokaryotic and fungal communities. The sampling was also done taking into account the distance from the roots of the plant, because the root exudates are reported to substantially influence the bacterial communities present in their vicinity compared to those present in the bulk soil (Philippot et al., 2013).

## 2. Materials and methods

### 2.1. Sampling

This study was conducted in a restricted geographical area to minimize pedoclimatic influences such as altitude, temperature, rainfall, humidity, wind, sun exposure, temperature excursion, soil slope or latitude and longitude (Bokulich et al., 2014). The Collio region is in fact a very heterogeneous hilly geographical area, both in terms of climate and soil composition, a characteristic that makes this region famous worldwide for the production of Collio D.O.C. wines (designation of controlled origin), characterized by a high qualitative and sensorial differentiation despite being produced in the same area. The sites of the two analyzed managements were therefore chosen after a careful selection to maintain all the environmental factors in the exact same conditions, condition that was not possible to maintain in other vineyards even in immediately adjacent areas Furthermore, the work was set up to give a real and concrete response to local producers, with the actual cultivation conditions implemented. Therefore, given the impossibility of standardizing conditions as would happen in a greenhouse experiment on which it would have been possible to apply other experimental designs, and given the fact that analyzing multiple plots located in places distant from each other would only increase the background noise leading to the observation of possible differences not induced by the treatment but by environmental conditions and soil composition, we implemented sampling with a clumped segregation design (Hurlbert, 1984).

Twelve sampling points were considered between these two adjacent vineyards of cultivar Merlot. Six sampling points concerned a vineyard managed with organic cultivation methods and six concerned a vineyard managed with conventional methods, both maintained for 10 years with the same agronomic practices. The vineyard was located within the Collio DOC area in Corno di Rosazzo (Italy), and sampling carried out during October 2020. Sampling was conducted considering the entire portion of the vineyard, equally sampling the plants present at the beginning, centre, and end of the rows. The different management protocols of these vineyards, including planting period, phytosanitary treatments, fertilization and agronomic practices, and sampling GPS coordinates are summarized in Table 1. For each sampling point, soil aliquots were obtained with the aid of a mini excavator at a depth of 50 cm, perpendicularly to the stem of the vine and intercepting the root system and the rhizosphere zone at three different distances from the plant roots: on the plant root (R), from the rhizosphere soil (VR), and from bulk soil without the presence of roots (GEN). The microbiological analyzes were carried out on soil kept moist at 5°C (for 24 h), while the chemical analyzes were carried out on a portion of dried and sieved soil.

**Table 1:** Summary of samples and their agronomic management differences between the conventional and organic vineyards in the sampling year.

	Organic	Conventional
Sample GPS coordinates	**C1** 46°00′25.937” N 13°24′49.275″ E	**C7** 46°00′26.290” N 13°24′44.260″ E
	**C2** 46°00′25.540” N 13°24′49.398″ E	**C8** 46°00′25.113” N 13°24′41.020″ E
	**C3** 46°00′25.183” N 13°24′49. 483″ E	**C9** 46°00′24.176” N 13°24′37.263″ E
	**C4** 46°00′27.471” N 13°24′45.282″ E	**C10** 46°00′25.667” N 13°24′44.340″ E
	**C5** 46°00′26.641” N 13°24′43.630″ E	**C11** 46°00′25.500” N 13°24′42.801″ E
	**C6** 46°00′27.240” N 13°24′40.966″ E	**C12** 46°00′25.330” N 13°24′40.984″ E
Vineyard history		
Vineyard age (years)	72	24
Years of organic management	10	/
Differences in phytosanitary treatments		
Cu usage	3.75 kg ha^−1^ year	1.85 kg ha^−1^ year
Other products	*Products approved for organic farming*: Natural pyrethrum, orange essential oil, seaweed extract, amino acids	*Chemical synthesis products*: acetamiprid, metrafenone, phosphite, mandipropamid, metiram, fluxapyroxad, fenbuconazole, zoxamine, oxathiapiprolin
Green manure		
Management	Alternate rows (Mix Brassicaceae, legume, grasses)	None
Fertilizations		
Products	Organic mix (1.5 t ha^−1^ year^−1^) Manure (2.0 t ha^−1^ year^−1^)	Organic mix (1.5 t ha^−1^ year^−1^) Manure (1.0 t ha^−1^ year^−1^)

### 2.2. Soil chemical analysis

For chemical analyses, 0.5 kg of the collected soil, air-dried for 48 h, were homogenized by sieving excluding particles with a diameter > 2 mm. For pH measurement, 10 g of soil were dissolved in 25 mL of deionized H_2_O and stirred for 15 min. After 30 min of rest, the values were measured using a pH-meter with glass-combined electrode (Basic 20, Crison Instruments, Spain).

Pseudo-total macro-, micro-and toxic elements were measured using the USEPA 3052 (USEPA, 1995) mineralization method and inductively coupled plasma atomic emission spectroscopy, ICP-AES (Agilent 5800). In brief, 0.5 g of soil was digested in a microwave oven using 10 mL of concentrated nitric acid. The digest solution was filtrated (<0.2 μm) and measured, after dilution and addition of scandium (Sc) as internal standard.

Total organic carbon and total nitrogen (Corg and Ntot) were determined by automated thermal analyses where carbon is converted to CO_2_ by flash combustion at 1080°C (MicroCube, Elementar). Carbonates were previously removed from 10 mg of soils by treatment with HCl in silver capsules, then calculating C/N ratios.

Carbonates have been measured volumetrically with a Scheibler calcimeter (Austrian Standards International, 1999. Önorm 1,084: Chemical analyses of soils—Determination of carbonate. Vienna, Austria.).

Soil microbial biomass C and N (Biomass-C and Biomass N) were measured by the fumigation-extraction method (Vance et al., 1987) on preincubated moist soils (25°C for 5 to 7 days). Briefly, three portions of moist soil, each containing 25 g oven dry soil, were fumigated with ethanol-free chloroform for 24 h. After the removal of chloroform, soil samples were transferred to 250 mL plastic bottles and extracted with 100 mL 0.5 M potassium sulfate (1:4 soil to solution ratio). A set of non-fumigated soils were extracted similarly. Organic C and N in the soil extracts were measured by liquid TOC-TN (Shimadzu VCPN). Soil microbial biomass C (Biomass-C) was calculated from: Biomass C = (extractable C in the fumigated sample) minus (extractable C in the unfumigated sample) divided by 0.45 (K_EC_). Biomass N was calculated from: Biomass N = (extractable N in the fumigated sample) minus (extractable N in the unfumigated sample) divided by 0.53 (K_EN_) (Joergensen, 1996).

Basal respiration was calculated from the CO_2_ trapped during incubation minus the blank (no soil) and divided by the incubation time (Anderson and Domsch, 1993). On the obtained data, the statistical analysis was carried out through R v4.1.2. After verifying the variance with F test and normality by Shapiro–Wilk, the significance was evaluated by T test, considering significant results with value of *p* < 0.05.

### 2.3. DNA sequencing

For DNA extraction, 10 g of soil sample added with 10 mL of sterile ultrapure water were homogenized in a sterile stomacher bag to remove coarse debris, centrifuged at 12000× *g* for 10 min to remove the water, and then dried using a vacuum concentrator (Concentrator 5301, Eppendorf, Germany). Genomic DNA was extracted from 0.25 g of dry medium using the DNeasy Power Soil Kit (QIAGEN, Germany). The quantity and quality of the purified DNA were determined by spectrophotometry at 260 nm using a NanoDrop 2000c (ThermoFisher Scientific, USA). For DNA metabarcoding analysis, 16S rRNA gene for bacteria and ITS for fungi were used as target regions. Libraries were prepared with the KAPA Hyper Plus Kit (Roche, Swiss). For the amplification of the V3-V4 region of the 16S the pair of primers 341F (5′-CCTACGGGNGGCWGCAG-3′) and 805R (5′-GACTACHV GGGTATCTAATCC-3′) were used (Hugerth et al., 2020), while for the amplification of the ITS1 region the primers pair ITS1 (5′-CTTGGTCATTTAGAGGAAGTAA-3′) and ITS2 (5′-GCT GCGTTCTTCATCGATGC-3′) were used (Op De Beeck et al., 2014). The samples were then sequenced in paired-end mode, with a length of 300 bp with the MiSeq platform (Illumina, USA). The obtained sequences were published on Sequence Read Archive (SRA) from the National Center for Biotechnology Information (NCBI) with the bioproject number PRJNA946685.

### 2.4. Bioinformatic analysis

After a preliminary quality check of the raw reads carried out through FastQC v0.11.9 (Andrews, 2010), the subsequent bioinformatics analyzes were conducted in the Qiime2 Release 2021.11 environment (Bolyen et al., 2019). The primers were trimmed using Cutadapt v3.5 (Bolyen et al., 2019), and through DADA2 v2020.11.0 (Callahan et al., 2016) the reads were re-checked for their quality, filtered and denoised. The pre-trained SILVA (Quast et al., 2013) v.138 99% OTUs full-length sequences Naïve Bayes classifier was used for the taxonomic analysis of the 16S reads, while for the analysis of the ITS reads the Unite (Nilsson et al., 2019) v8.3 dynamic 2021-05-10 database with all eukaryotic species was used for training the Naïve Bayes classifier. From the elaborations obtained, the reads belonging only to bacteria and fungi, classified at least at the phylum level, and deprived of singletons were extrapolated. The subsequent statistical analyzes were conducted in R v4.1.2 environment using the Supplementary Table 1 as input metadata, taking in account the different treatment (Treatment), the distance from the plant roots (Site) and their interaction (SiteGroup). The rarefaction curves on species richness were calculated using ggrare from ranacapa package (Kandlikar et al., 2018). After rarefaction, on the samples implemented through the phyloseq package, the Alpha-diversity analysis was carried out. Significant effects were tested using a pairwise Kruskal-Wallis H test considering significant results at value of *p* < 0.05. Using the vegan package (Oksanen et al., 2013), a PcoA was used to visualize the distribution pattern of the microbial and fungal populations. Through the adonis function of the vegan package, a PERmutational Multivariate Analysis of Variance was also carried out, making 4,999 permutations on the rarefied ASVs in order to study the effect of the Treatment and of the Site. To understand how microbial populations correlated with environmental data, a distance-based redundancy analysis (dbRDA) was performed using the vegan capscale function. The reconstruction of metabolic functions was made with FAPROTAX v 1.2.4 (Louca et al., 2016). The graphical representations of the results were plotted in R using the ggplot2 library (Wickham, 2016).

## 3. Results

### 3.1. Environmental data

**Figure 1:**
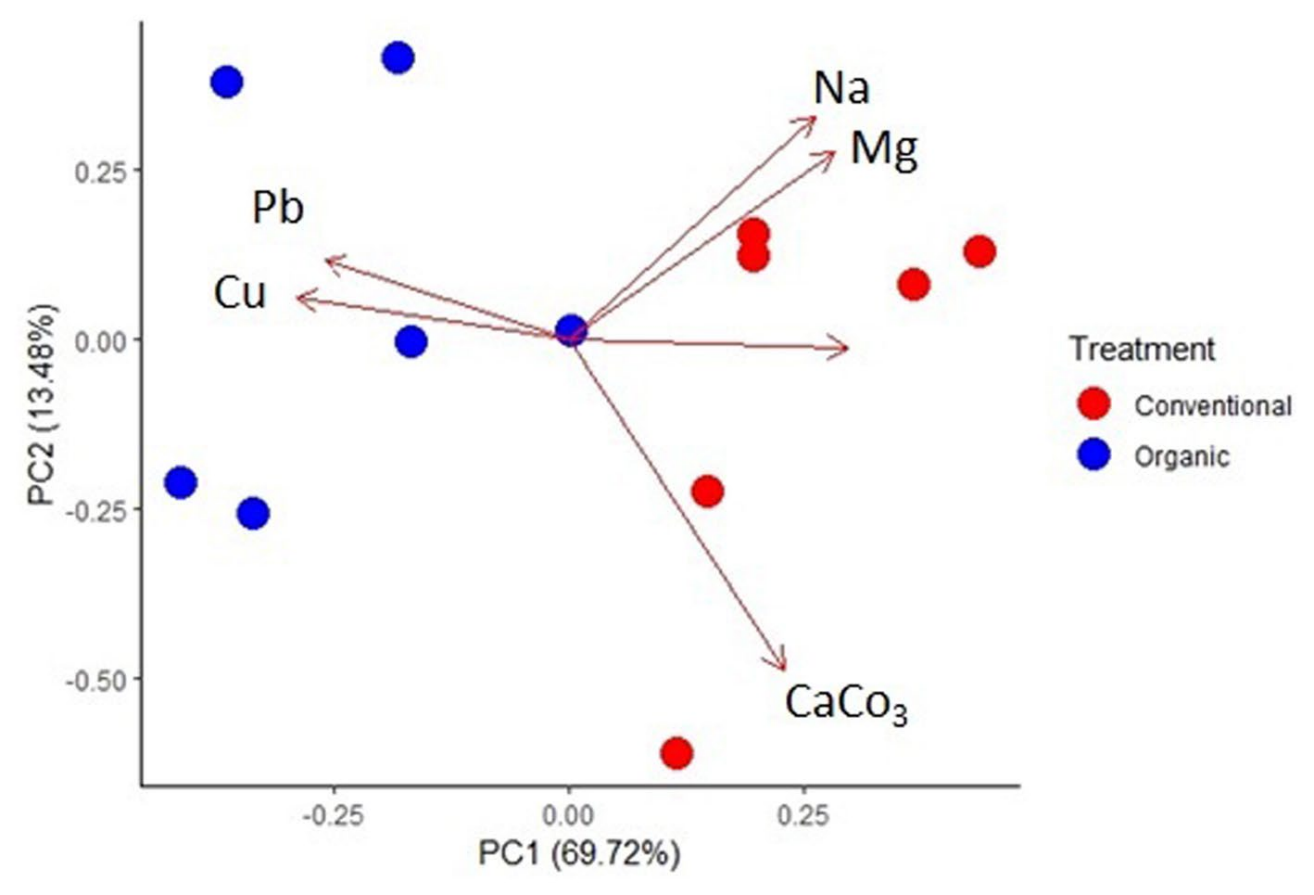
PCA analysis representing the relation between chemical parameters significantly different between the two treatments, in relation to the different sampling points.

The average values and the significance of each environmental variable, compared in relation to the Organic and Conventional treatments were reported in Table 2. As regards the content of microelements, it was possible to detect significant differences at a statistical level between the two treatments only for Cu. This element was present at a higher concentration (*p* < 0.01) in the Organic treatment, with an average value of 146.10 ± 34.97 μg/g, compared to the Conventional treatment which showed an average value of 79.46 ± 18.46 μg/g. Furthermore, analyzing the microelements it was observed a significantly higher concentration (*p* < 0.05) of Mg (5030.80 ± 548.83 μg/g for Organic; 5769.67 ± 550.06 μg/g for Conventional), and Na (135, 47 ± 20.88 μg/g for Organic; 189.97 ± 41.67 μg/g for Conventional) in the Conventional treatment. Although the average concentrations of Ca, K, P, S and Al were generally higher in the Conventional treatment, their difference was not statistically significant. Considering the potentially toxic elements, only Pb was significantly higher (*p* < 0.05) in the Organic treatment (24.33 ± 4.22 μg/g) compared to Conventional (19.01 ± 1.00 μg/g). Regarding other parameters, CaCO_3_ showed a significant difference (*p* < 0.01) between the two vineyards, with higher values for Conventional (46.03 ± 3.89 μg/g) than for Organic (37.29 ± 4.55 μg/g). These results also correlated with significantly different (p < 0.05) pH values, that resulted higher for the Conventional (7.95 ± 0.12) in comparison to Organic (7.73 ± 0.23) soil. Through the Principal Component Analysis (PCA) reported in Figure 1, it was possible to represent these results, visualizing how the different sampling points were arranged based on chemical parameters which were statistically different between the two treatments (*p* < 0.05). In fact, it was possible to observe a clear separation between the Conventional soil samples, which were more associated with higher values of Na, Mg, CaCO_3_ and pH, compared to the Organic soil samples, associated with higher concentrations of Cu and Pb.

**Table 2:** Soil chemical properties.

Variable	Organic		Conventional		Value of *p*
	Mean	SD	Mean	SD	
General parameters					
pH	7.82	0.02	7.97	0.05	*
CaCO_3_	37.29	4.55	46.03	3.89	**
Basal resp.	18.45	3.02	17.09	2.97	
Corg	1.46	0.34	1.17	0.34	
Ntot	0.14	0.03	0.15	0.04	
Biomass C	203.35	62.66	249.51	15.91	
Biomass N	62.08	13.71	68.05	34.18	
Macro-elements					
Ca	17985.48	8825.15	26401.93	3694.74	
Mg	5030.80	548.83	5769.67	550.06	*
Al	26061.67	1618.31	27945.43	1769.84	
Na	135.47	20.88	189.97	41.67	*
K	4017.17	479.04	5127.00	1286.82	
P	480.04	158.13	546.52	220.07	
S	202.99	32.92	279.66	105.44	
Micro-elements					
Cu	146.10	34.97	79.46	18.46	**
Zn	89.17	4.62	92.71	6.93	
B	15.82	2.59	18.72	4.93	
Mn	1615.63	349.65	1275.34	184.23	
Fe	33813.08	2460.70	33626.38	1046.14	
Toxic elements					
Co	15.89	2.69	14.82	1.15	
Cr	58.77	5.66	64.08	9.56	
Ni	78.67	5.79	82.22	4.39	
Pb	24.33	4.22	19.01	1.00	*
Cd	0.92	0.08	0.92	0.04	

Statistical analysis (*T* test) is reported (*p < 0.05, **p < 0.01). Total carbonates CaCO_3_ were expressed as g kg^−1^; Basal resp. as mg CO_2_ g^−1^ soil h^−1^; Corg and Ntot as g kg^−1^; Biomass C and Biomass N as μg g^−1^; Macro- Micro-and Toxic elements (Ca, Mg, Al, Na, K, P, S, Cu, Zn, B, Mn, Fe, Co, Cr, Ni, Pb, Cd) as μg g^−1^ of soil.

### 3.2. Sequencing results

Through the sequencing process, 4,413,339 reads were obtained from the sequencing of 16S regions for bacterial population, while 4,442,942 raw reads were obtained from the sequencing of the ITS region of the fungi population, that following the denoising operation carried out via DADA2, resulted in 2,482,232 reads for the 16S and 2,411,200 reads for the ITS. After the removal of the reads unclassified at least at the phylum level and the reads present with a frequency *n* < 2 (singletons), 17,292 features with a total frequency of 1,884,598 for the 16S, and 2,519 features with a total frequency of 998,124 for ITS were observed. From the plot of the rarefaction curves reported in Supplementary Figure 1, it can be seen how the sequencing effort was sufficient to analyze the biodiversity of the samples, as all the curves reached the plateau.

### 3.3. Alpha diversity

**Figure 2:**
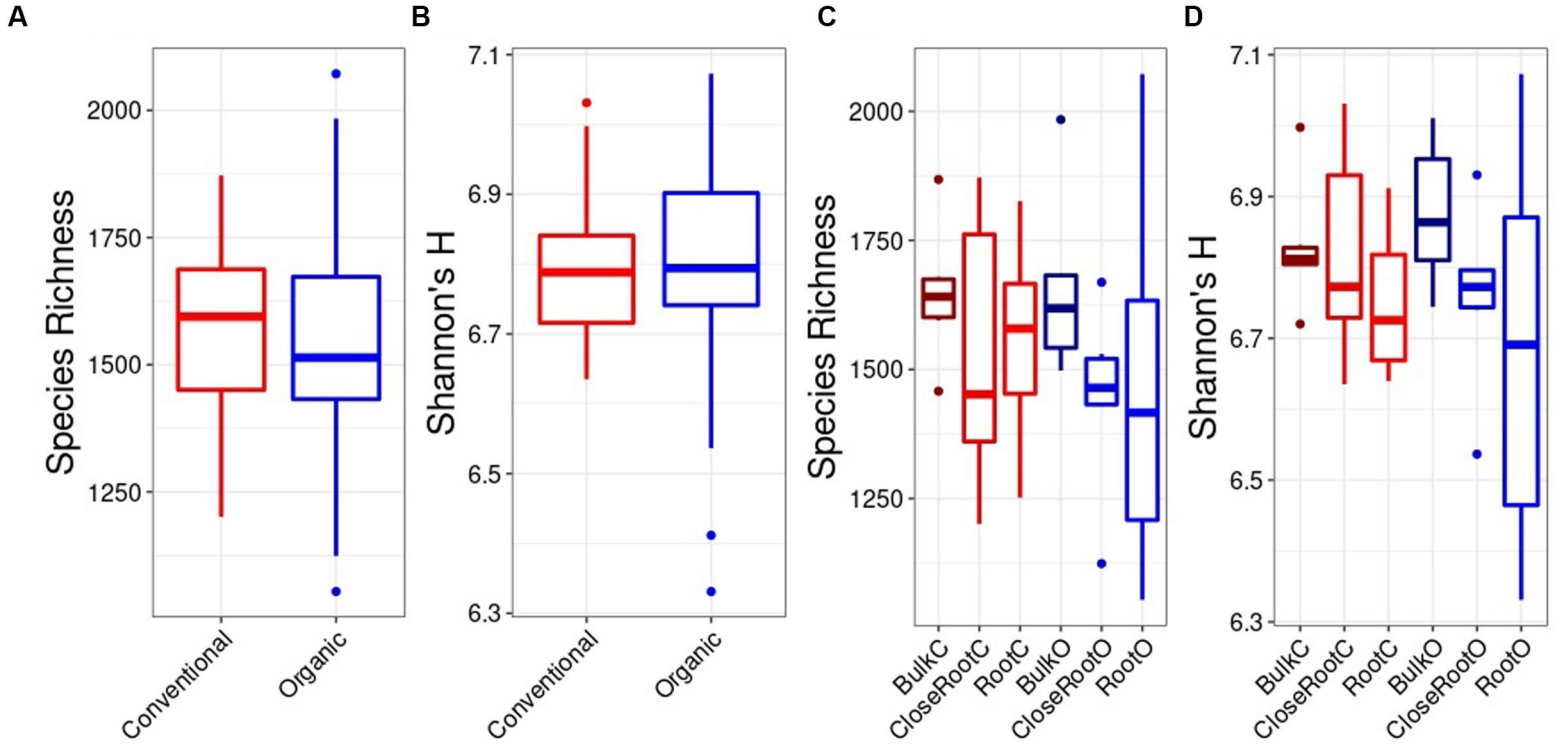
Bacteria alpha diversity evaluated in relation to the SiteGroup (Species Richness **A**, Shannon’s H **B**) and Treatment (Species Richness **C**, Shannon’s H **D**) represented as boxplots.

The alpha diversity between samples was investigated using the Species Richness and Shannon’s H indexes. Considering the differences brought by the SiteGroup using the Kruskal Wallis test for bacterial community, no significant differences were identified either for the Species Richness (Figure 2A) or for the Shannon index (Figure 2B). Furthermore, since there were no significant differences within the two Treatments according to the Site, it was possible to the Kruskal Wallis test for bacterial community, no significant differences were identified either for the Species Richness (Figure 2A) or for the Shannon index (Figure 2B). Furthermore, since there were no significant differences within the two Treatments according to the Site, it was possible to compare the alpha diversity indices overall between the samples belonging to the Conventional and Organic treatment. Analyzing the species richness, i.e., evaluating only the number of species, the value of this index was higher in the Conventional treatment (1577.56 ± 26.93) than in the Organic treatment (1523.67 ± 262.61). Similarly, accounting both for abundance and evenness of the taxa, the Conventional treatment showed values of the Shannon index (6.80 ± 0.12) higher than the Organic treatment (6.77 ± 0.20). However, also in this case, analyzing the samples based on the two treatments using the Wilcoxon test, there were no significant differences (*p* > 0.05; Figures 2C,D).

**Figure 3:**
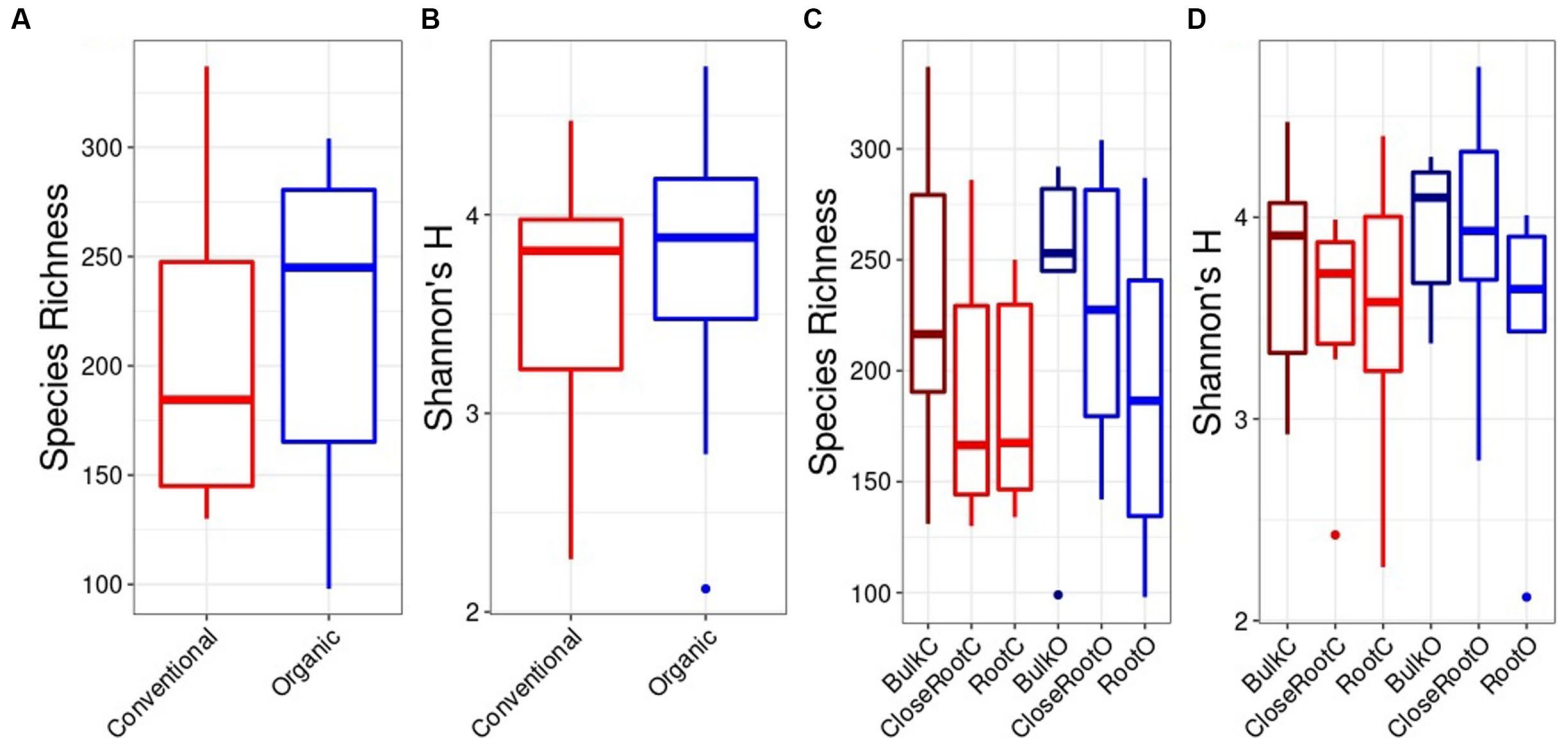
Fungi alpha diversity evaluated in relation to the SiteGroup (Species Richness **A**, Shannon’s H **B**) and Treatment (Species Richness **C**, Shannon’s H **D**) represented as boxplots.

The same dynamics were observed for the fungi population. In fact, no significant differences were found for alpha diversity in relation to the SiteGroup both by evaluating the species richness and the Shannon index (Figures 3A,B). Also in this case, since there were no significant differences made by the Site within the Treatments, it was possible to compare the Conventional and Organic treatments as a whole. In this case, the Organic treatment showed higher values of species richness (218.28 ± 69.48) and Shannon index (69.48 ± 0.61) in comparison to the Conventional treatment (201.56 ± 3.58 for species richness, 63.71 ± 0.62 for Shannon’s H). However, even in this case, it was not possible to detect significant differences using the Wilcoxon test (*p* > 0.05) (Figures 3C,D).

### 3.4. Beta diversity

**Figure 4:**
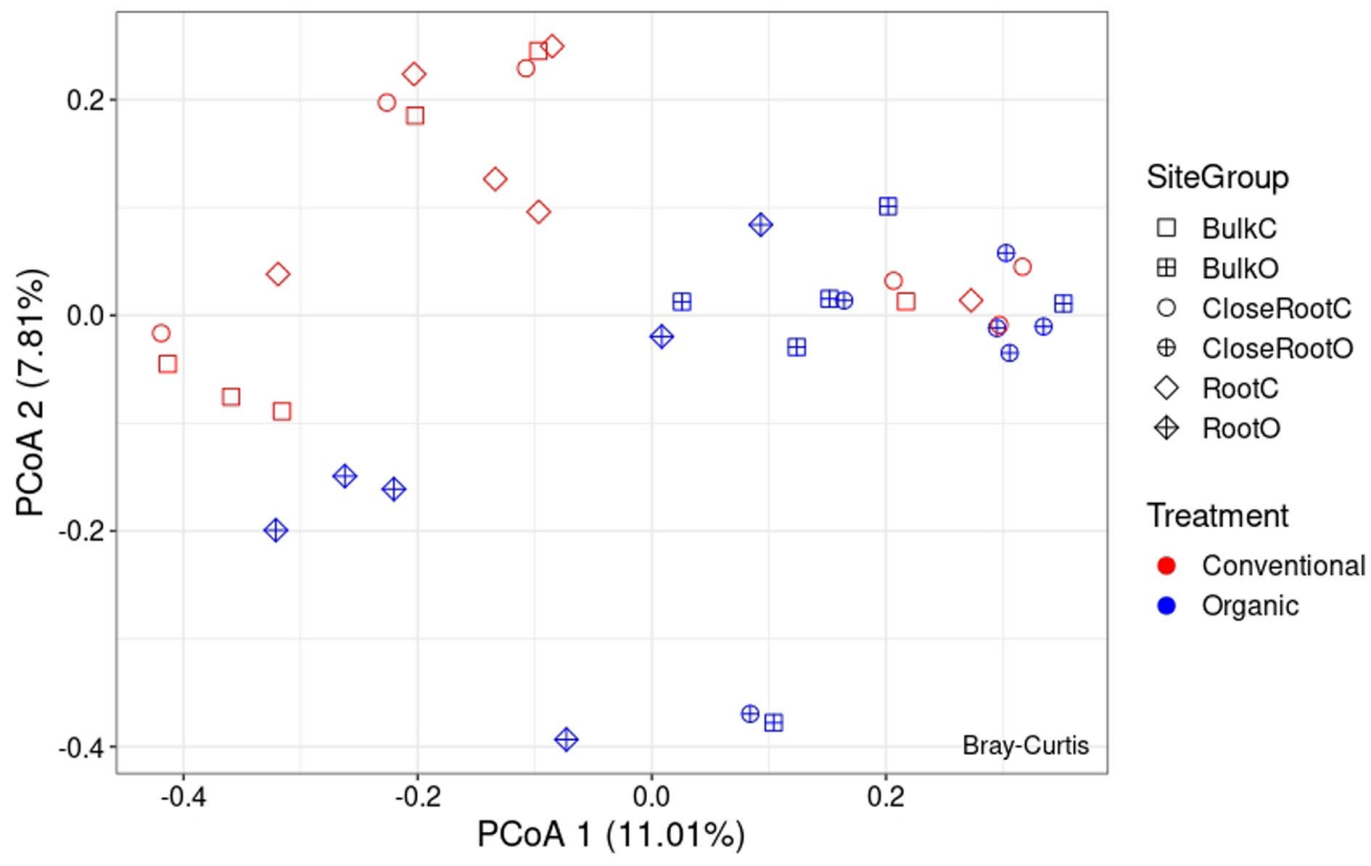
Principal Coordinate Analysis (PcoA) based on Bray–Curtis dissimilarity in prokaryotic community.

**Figure 5:**
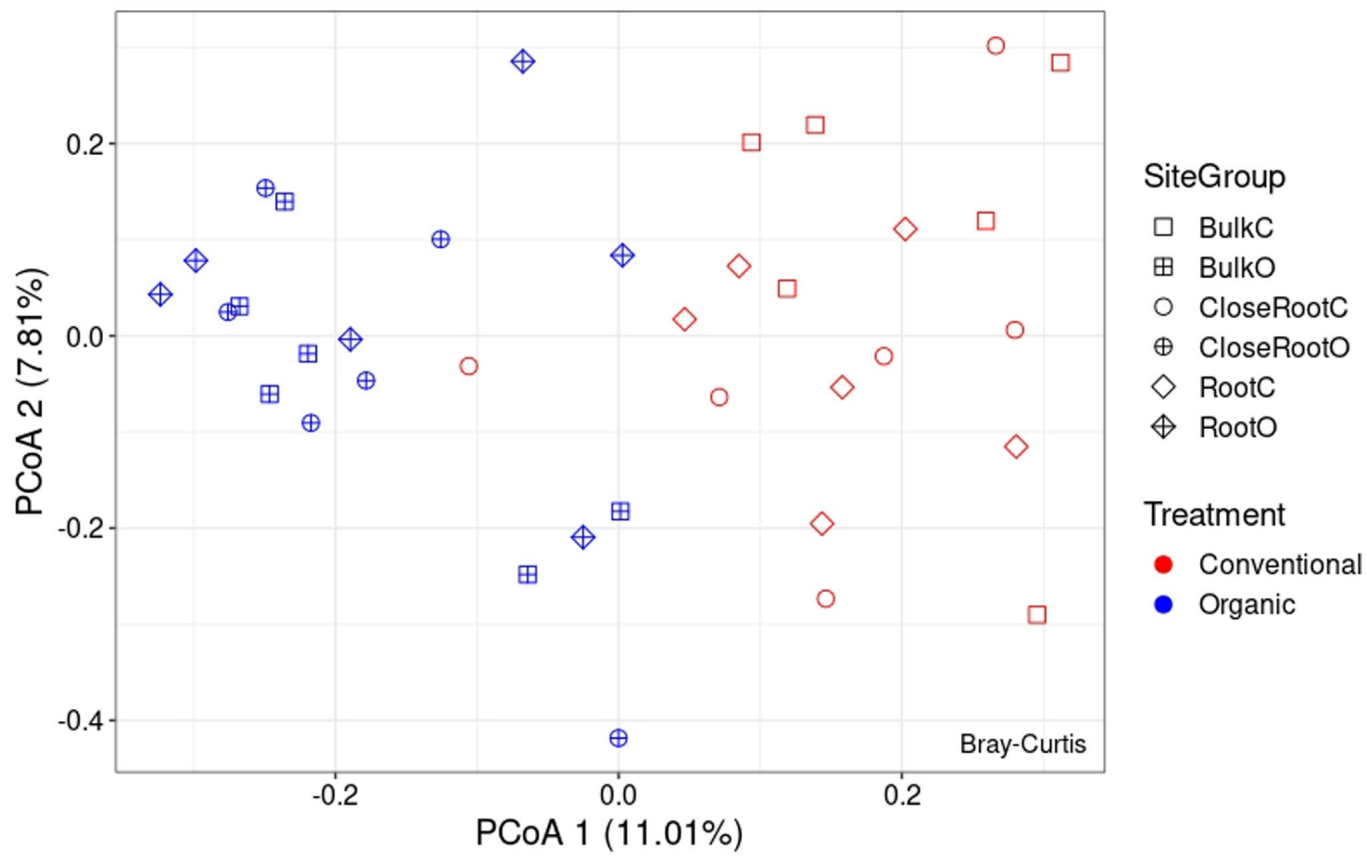
Principal Coordinate Analysis (PcoA) based on Bray–Curtis dissimilarity in fungal community.

Differences in the microbial communities among the two treatments were assessed using the Bray–Curtis dissimilarity. As regards the bacterial population, from the PcoA reported in Figure 4 it was possible to observe the results brought to light by PERMANOVA on the significance of the considered variables. As reported in Supplementary Table 2, Treatment (*p* < 0.01) and Site (*p* < 0.05) resulted significant in shaping the prokaryotic community, while the result of the interaction between the two (SiteGroup) was not significant. However, given the low values of R^2^ the reconstructed model was not very effective in explaining the variance (as can be seen, Treatment explained ∼8.8% of the variance and Site ∼8.6%, and the total variance explained by all considered factors was ∼23.7%). Also with regard to ITS whose PcoA is reported in Figure 5, the PERMANOVA results once again highlighted the significance of the Treatment (*p* < 0.01) in the shaping of the fungal community. However, even in this case, as can be seen in Supplementary Table 3, although significant, given the low R^2^ value of the model, the Treatment explained only ∼9.0% of the variance.

### 3.5. Relations of soil microbiota with soil chemical properties

**Figure 6:**
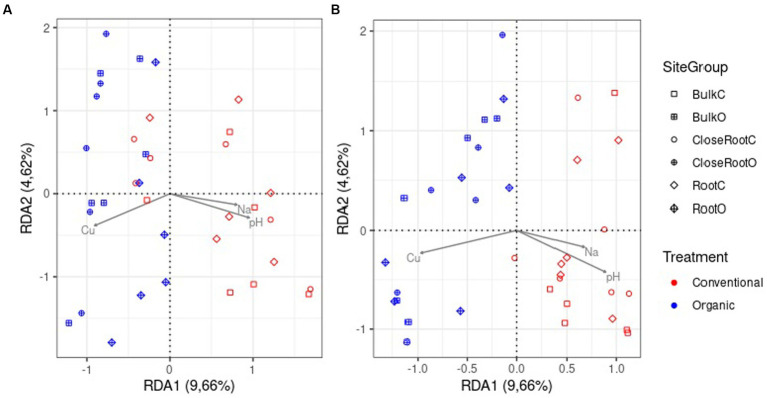
Distance-based redundancy analysis (dbRDA) based on Bray–Curtis dissimilarity in community composition for prokaryotic **(A)** and fungi **(B)** communities.

To analyze the relationship between soil chemical properties and prokaryotic and fungal communities, a variance partitioning analysis and dbRDA were performed on a reduced model in order to avoid variables correlation, considering only the factors Cu, pH and Na. The variance partitioning analysis showed that these three parameters together explained 7.6% of the variance for prokaryotic and 7.7% of the variance for fungi population (Supplementary Table 4). The dbRDA (Supplementary Table 5) highlighted a significant role (value of *p* < 0.01) of Cu in shaping both the bacterial and fungal communities. pH also contributed significantly to both communities, however more significantly in the fungal (value of *p* < 0.01) than in the prokaryotic community (value of *p* <0.05). Conversely, the contribution of Na was not significant (value of *p* > 0.05). In Figure 6 the results obtained from the dbRDA for the 16S (A) and ITS (B) were graphically represented. It was possible to note how Cu and pH effected the composition between the two treatments, with a clear separation especially in fungal community.

### 3.6. Taxonomic composition

**Figure 7:**
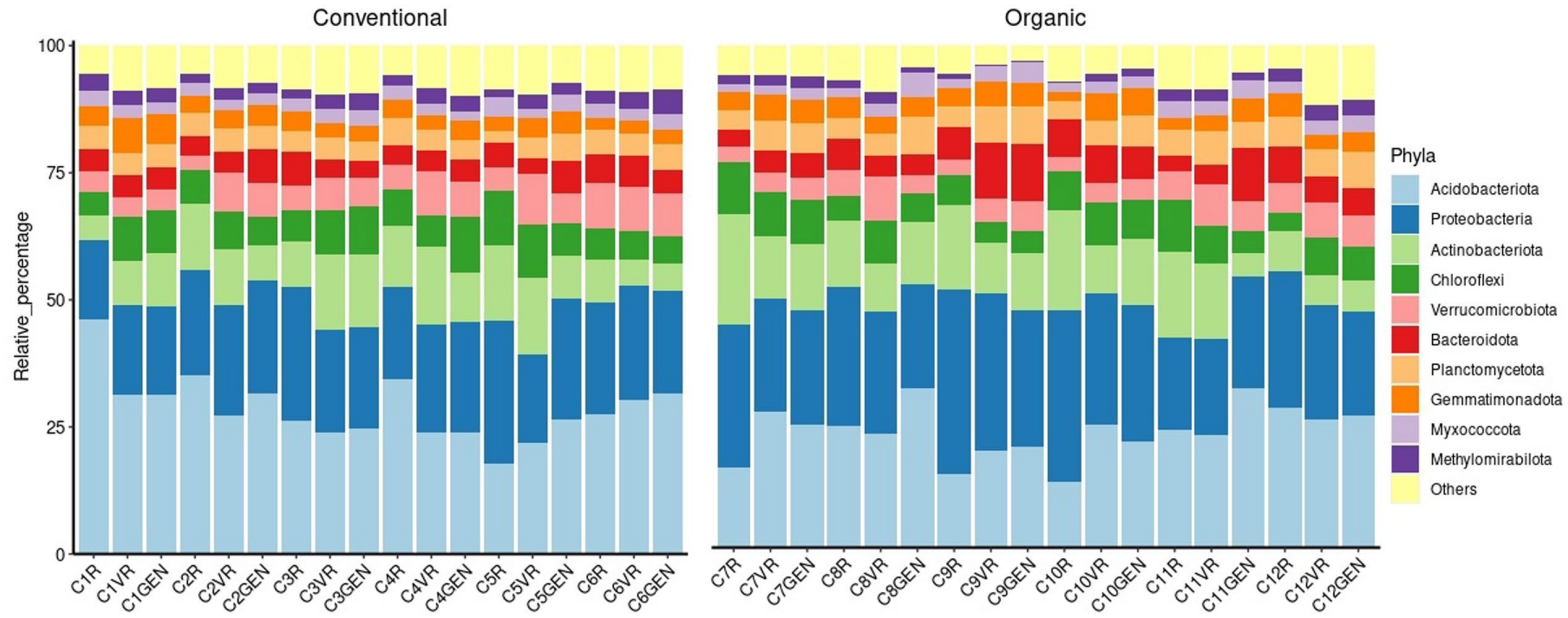
Prokaryotic composition at phylum level.

Through to the conducted analyses, it was also possible to verify how the considered microbial populations were influenced from the two treatments. From Figure 7, representing relative percentages of the 10 major phyla, it was possible to observe the structure of the prokaryotic populations of the samples. Overall, *Acidobacteriota* was the phylum present in higher percentages with a mean relative abundance of 25.89%, followed by *Proteobacteriota* with a mean relative abundance of 22.20%, representing combined almost half of the identified phyla. By analyzing the data reported in Supplementary Table 6, it was possible to find significant differences for 15 phyla. In fact, in the same most representative groups, it was possible to observe a significantly higher presence of *Proteobacteria* in the conventional treatment, while a higher level of *Acidobacteriota* characterized the organic treatment. Other major groups that increased in conventional treatment included *Bacteroidota* and *Planctomycetota*. Considering the phyla at lower percentages, conventional treatment was characterized by a significantly higher presence of *Patescibacteria*, *Elusimicrobiota*, *Sumerlaeota*, *Fibrobacterota*, WS2, and *Deinococcota*, while the organic treatment showed higher percentages of *Methylomirabilota*, NB1-j, *Desulfobacterota*, and MBNT15.

**Figure 8:**
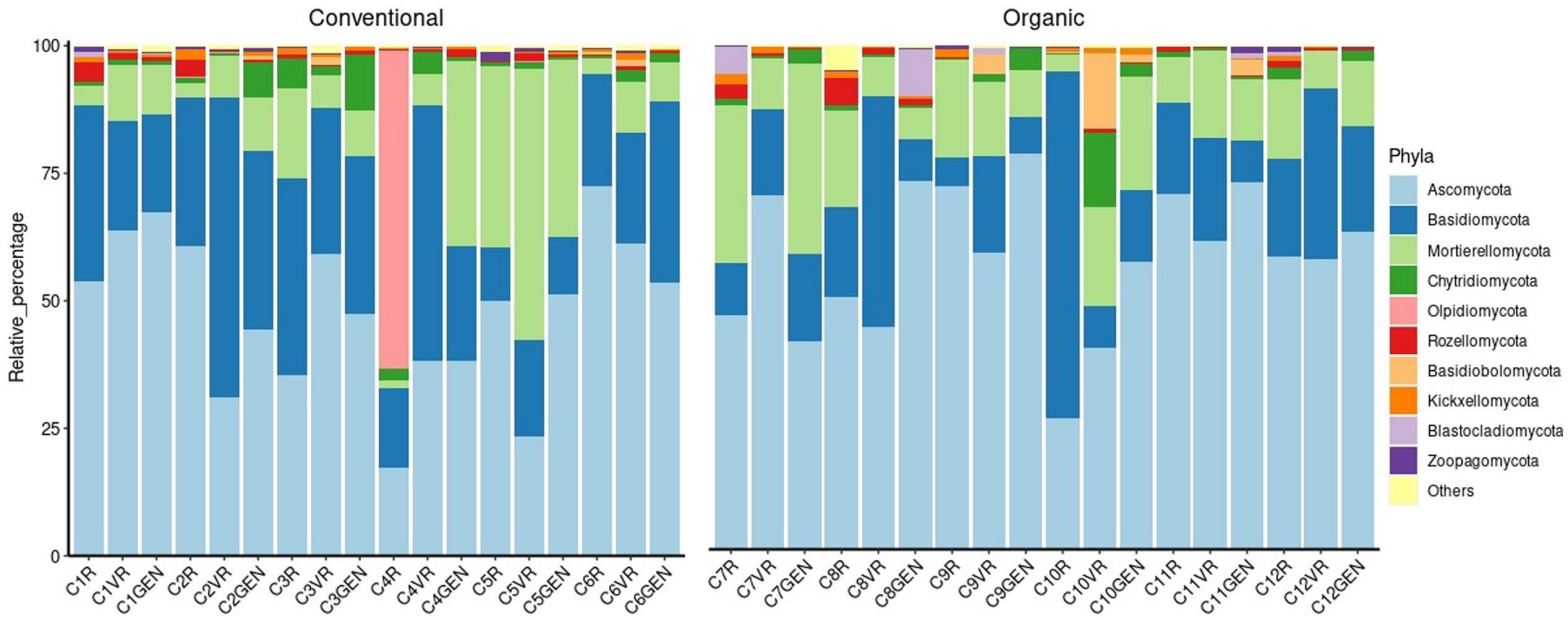
Fungi composition at phylum level.

Considering the fungal population, as can be seen from the Figure 8, it was observed a different distribution in comparison to the prokaryotic one. In this case, in fact, the phylum *Ascomycota* present with an average relative abundance of 55.34%, alone constituted more than half of the phyla present, while the second most present phylum was *Basidiomycota* with a median relative abundance of 19.98%. In addition, the presence of fungal species, which constitute the majority of some samples but are not present at significant levels in the other samples, was observed. For example, the sample C4R taken near the root system of the plant, was characterized by the presence of a fungus belonging to the genus *Olpidium* (62.46%), a zoosporic pathogen of plants, animals, fungi, and oomycetes. Also, samples taken at point C7 were characterized by the high presence of fungi belonging to the genus *Leucoglossum* constituting 51.49% of the reads in the sample C7VR, 15.67% in C7R, and 7.06% in C7GEN. Other examples were in sample C3R highly characterized by the presence of *Armillaria borealis* (24.12%), the presence of *Psathyrella* spp. in samples C2VR (39.16%) and C2GEN (12.93%), *Phaeomonillea chlamydospora* in sample C9R (19.58%), and *Alfaria* spp. in sample C12VR (25.64%).

Due to such a high internal variability between samples, the cases in which the differences were significant were few (Supplementary Table 7). The main difference concerned the *Ascomycota*, which were present in higher percentages in the conventional treatment (57.85%) than in the organic treatment (48.28%). The second significant difference concerned the *Entorrhizomycota*, in this case present in higher percentages in the organic treatment (0.09%) than in the conventional treatment (0.02%).

### 3.7. Metabolic function differences

**Figure 9:**
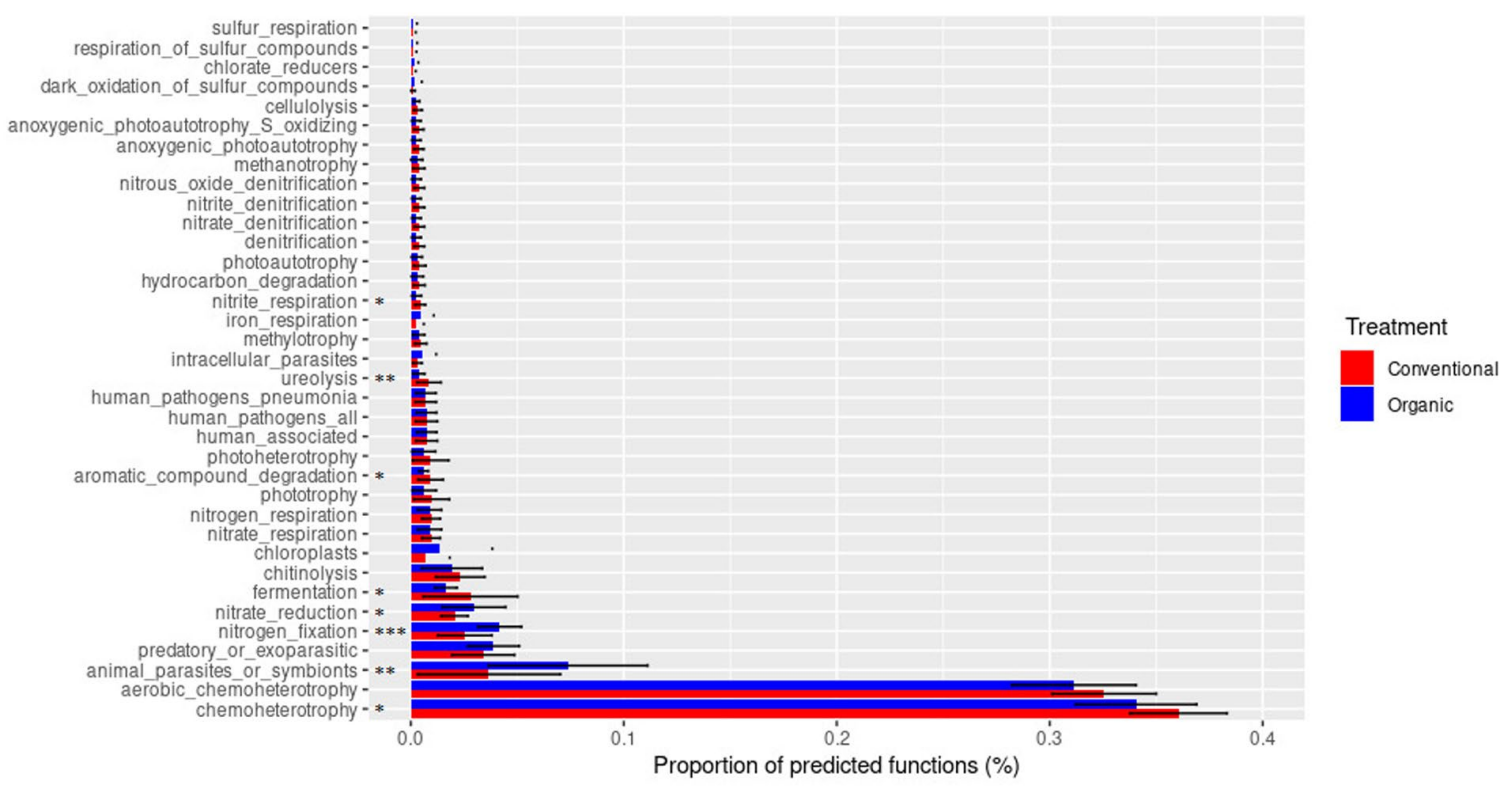
Metabolic and ecologically relevant functions of prokaryotic clades reconstructed through the FAPROTAX database. Statistical analysis (T test) made on the percentage of the predicted function is reported (*p < 0.05, **p < 0.01 ***p < 0.001).

Using the FAPROTAX database, it was possible to analyze the metabolic and ecologically relevant functions of the prokaryotic clades (Figure 9). It can be seen that the main functions found were mainly related to chemoheterotrophy metabolisms, identified in significantly higher percentages in the conventional treatment, and specifically aerobic chemoheterotrophy. Other functions significantly more present in the conventional treatment were nitrite respiration, ureolysis, aromatic compound degradation, and fermentation. On the contrary, the organic treatment was significantly characterized by nitrate reduction and nitrogen fixation.

## 4. Discussion and conclusion

The soil chemical parameters revealed significant differences between the two treatments for some of the considered parameters. Higher concentrations of Cu were measured in the organic managed soil, as would be expected given the higher use of copper-based compounds as phytosanitary treatment. In fact, this compound is one of the most important active ingredients of fungicide products used in organic agriculture, and its use can lead to its accumulation in the soil (Pellegrini et al., 2021; Tamm et al., 2022; Jez et al., 2023). Surprisingly, the Organic treatment was also linked to a higher concentration of Pb in the soil. In the literature, an increase in Pb concentration due to the use of livestock manure (Kumar et al., 2013) or of some phytosanitary treatments (Merry et al., 1983) has been reported. However, the same organic manure was used in both treatments, and the employed phytosanitary treatments did not contain Pb. So, in our opinion, given the low levels when compared to the average levels of natural soil contamination (Bindler et al., 1999) and other comparable agricultural sites (Beone et al., 2018), their minimum differences, albeit statistically significant, can be attributed to minimal differences in the soil rather than from the different treatments performed. It should also be noted that Pb, along with Cu and all the other potentially toxic compounds, were found to be well below the Italian legislation threshold, which sets contamination levels for agricultural soils at 200 mg/kg for Cu and 100 mg/kg for Pb (Gazzetta Ufficiale della Repubblica Italiana, 2019, Art. 3). Even the other elements regulated by Italian law such as Zn and Co, Cr, Ni and Cd, which did not show significant differences between the two treatments, were found to be well below the legislative thresholds, indicating a general good condition of the soil. Instead, differences in Na and Mg concentrations were discovered. These compounds were present at higher levels in conventional treatment, a phenomenon also observed in other studies (Zarraonaindia et al., 2020). This difference can be ascribed to the use of mineral fertilizers in previous years, whose contribution may have increased their soil concentration (Nemera et al., 2018). This observation can also be extended to the other analyzed macroelements, which had higher average concentrations in conventional treatment but were not statistically different (Pogrzeba et al., 2018). Another significant difference found in the soil between treatments was the higher pH values in the conventional treatment. This observation was also related to the higher CaCO_3_ content in the conventional soil, which could be the cause (Magdoff and Bartlett, 1985) and to higher Corg (about +25%) albeit not statistically significant. In contrast, no significant differences were found for bioindicators such as basal respiration, biomass C, biomass N, and factors such as Corg and Norg. The company’s care to soil agronomic practices to either conventional or organic vineyards can be traced back to the scarcity of differences found in the chemical–physical characteristics of the organic soil compared to the soil cultivated with conventional agronomic methods, in contrast to the widespread differences reported in the bibliography (Angelova et al., 2013). Indeed, as described in the materials and methods section, for the last 10 years the conventional vineyard has been fertilized with products allowed for organic farming, potentially reducing the differences between the two vineyards in particular regarding the soil organic matter. The following metagenomic analysis took into account the significantly different soil parameters between the two treatments, given the ability of factors such as Cu (Fagnano et al., 2020), Pb (Liu et al., 2007), Mg (Nicolitch et al., 2019), Na (Yan et al., 2015), and pH values (Zhou et al., 2017) to modulate the microbial population of the soil. There were no statistically significant differences in the number of bacterial and fungal species between the two treatments or the sampling point based on distance from the root (SiteGroup). This implied that any chemical–physical difference between the treatments had no effect on species richness. In particular, it can be deduced that the higher concentration of Cu due to the organic agronomic treatments had no significant impact on biodiversity levels, especially on the fungal one, which is particularly sensitive to the levels of this element (Bonanomi et al., 2016). However, as observed in the beta diversity analysis, the PERMANOVA results revealed differences in the composition of the microbial populations, particularly in relation to the treatment, while also in this case the SiteGroup was not significant. Although it had a significant impact on the shaping of microbial communities, the treatment could only explain a small amount of variation observed between samples, as seen in the reconstructed representations via PCoA. This result could be explained by the samples’ low variance due to the high conservation of the microbial groups present. In fact, all of the samples revealed a core of conserved species, which had previously been discovered in other similar studies on the soil of Italian vineyards (Coller et al., 2019). However, despite a general uniformity of the terrain, significant differences in the relative percentages of some phyla were observed, possibly indicating differences between the two treatments. In terms of the prokaryotic community, the most significant difference was observed in *Proteobacteria*, the main phylum of the conventional treatment, and *Acidobacteriota*, the main phylum of the organic treatment. This observation was consistent with other studies in the literature, which show that *Proteobacteria* are more associated with tilled agricultural soils, whereas *Acidobacteriota* are associated with forested soils and lower pH values (Kim et al., 2021). The ratio between these two indicator microorganisms can therefore suggest that the biological management has resulted in a condition more similar to that of a natural environment than conventional management methods. Other studies reported a positive correlation between *Proteobacteria* and *Patescibacteria*, and a negative correlation between *Methylomirabilota* and the level of mineralization of soil organic carbon (Dong et al., 2021). This phenomenon was also observed in this study, with a significantly higher presence of *Proteobacteria* and *Patescibacteria* in the conventional treatment, and a significantly higher presence of *Methylomirabilota* in the organic treatment, implying a possible correlation between the greater availability of organic carbon that had not yet been mineralized in the organic treatment. Differences have also been discovered for *Planctomycetota*, which are reported to be sensitive to the type of fertilization used (Buckley et al., 2006), and in the phylum NB1-J, whose functions are not yet clear, but the frequency of which has been reported to decrease in cultivated soil (Maretto et al., 2022). *Desulfobacterota* (as well as the related candidate phylum MBNT15) were found in higher concentrations in the organic treatment. Given their type of metabolism linked to the presence of organic matter, this could indicate a higher presence of the latter mentioned into the soil (Vipindas et al., 2022). Noteworthy was the exclusive presence of *Deinococcus* in the conventional treatment. In particular, this group of extremophile microorganisms (Slade and Radman, 2011) was discovered to be dependent on soil aeration (Ren et al., 2021). *Elusimicrobiota* were found at higher percentages in conventional, and their presence was reported to be related to higher CaCO_3_ concentrations (Constancias et al., 2015). In terms of fungi, there was a significant decrease in *Ascomycota* (which remained the main group) in the organic treatment, compared to a strong increase in *Basidiomycota*, which was not statistically significant. The increase of *Basidiomycota* in the organic treatment related to the application of organic fertilizer corresponds to what several authors reported (Dong et al., 2021; Xu et al., 2022). Another significant difference was identified with regard to the *Entorrhizomycota*, fungi related to the root system (Bauer et al., 2015) which were found to be more prevalent in the organic treatment in this test. Based on these findings, it was possible to conclude that there were no substantial differences in the core microbiota phyla ratio between organic management with low use of copper-based phytosanitary products and conventional management with fertilization using products permitted in organic farming, albeit with differences. In particular, no dysbiosis or reduction in biodiversity was observed due to the higher concentration of Cu in the soil, which was one of the main differences of the soil between the two samples studied (Mackie et al., 2013).

The FAPROTAX database analysis also revealed a conservation of bacterial functions between the two treatments, albeit with differences for some of them. Through this program that associates bacterial taxa with metabolic or ecologically relevant functions, which has been reported as valid in the literature for the analysis of soil functions (Sansupa et al., 2021), it was in fact possible to investigate any differences between the two treatments. The most notable distinction has been identified in terms of nitrogen fixation, which was more prevalent in the organic treatment. This could be explained in part by the larger use of organic fertilizer (Shi et al., 2021), but it could also be explained by the planting of legumes as green manure (Toda and Uchida, 2017; Table 1). Another difference found in agreement with other studies was the presence of a minor ureolysis function in the organic treatment (Fernández-Calviño et al., 2010). Other differences were discovered in nitrite respiration, fermentation, nitrate reduction, and chemoheterotrophy, which may indicate a difference in the availability of metabolic substrates and oxygen in the soil.

In conclusion, despite the phytosanitary treatments in organic vineyards caused more Cu accumulation in the soil than conventional management, it resulted not responsible for negative changes in microbial populations or the loss of biodiversity. In fact, when two soils were compared with the only significant difference being the phytosanitary protocols, the treatment was not identified as being responsible for high levels of induced variability. The concern that looms over the use of copper-based phytosanitary products about their accumulation and associated decrease in environmental biodiversity (Karimi et al., 2021), was not supported by these results. This suggests that with the doses applied in these treatments, Cu employment is compatible with the vineyard management without causing an impact on the soil microbiota, resulting in dysbiosis that could have a negative effect on vine’s health and productivity. These results could also be attributable to the use of organic fertilizers, in particular manure, that itself contains a great microbial biodiversity. Nonetheless, differences in the proportions of microbial populations can be used as indexes to confirm the treatment’s efficacy. Indeed, in the organic treatment, species more akin to non-cultivated environments have been promoted, and the merits could be attributable to the beneficial practices of this agricultural management approach.

## Data availability statement

The datasets presented in this study can be found in online repositories. The names of the repository/repositories and accession number(s) can be found in the article/Supplementary material.

## Author contributions

EC, LI, and MCi: conceptualization. EC, LI, MCo, and MCi: resources. AC, LI, MCo, and MCi: methodology. LI and MCi: supervision. AC and MCo: formal analysis, data curation, and writing – original draft. AC, MCi, and LI: visualization. LI: writing – draft revision. MCi and LI: writing – review and editing. LI and MCi: funding acquisition. All authors contributed to the article and approved the submitted version.
