# Supplementary material for: Organic vs. conventional: impact of cultivation treatments on the soil microbiota in the vineyard
Source: Front Microbiol. 2023 Oct 12;14:1242267. doi: 10.3389/fmicb.2023.1242267 (PMC10602642; doi:10.3389/fmicb.2023.1242267)
Supplement: Supplementary file 1 [file Data_Sheet_1.docx]

Supplementary Material

Organic vs Conventional: impact of cultivation treatments on the soil microbiota in the vineyard

Andrea Colautti, Marcello Civilini, Marco Contin, Emilio Celotti, Lucilla Iacumin^*^

*** Correspondence:**Lucilla Iacumin

Department of Food Science,

via Sondrio 2/A, 33100 Udine, Italy,

tel: +390432558126, Fax: +390432558130,

E-mail: [lucilla.iacumin@uniud.it](mailto:lucilla.iacumin@uniud.it)

**Supplementary figures**

**Supplementary Figure 1.** Rarefaction curves of 16S (A) and ITS (B) sequencing.

**A**
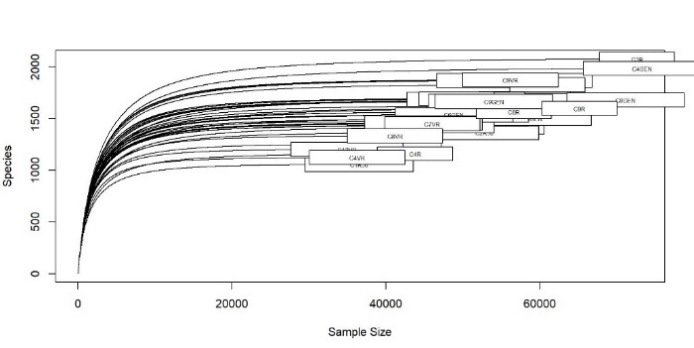


**B**

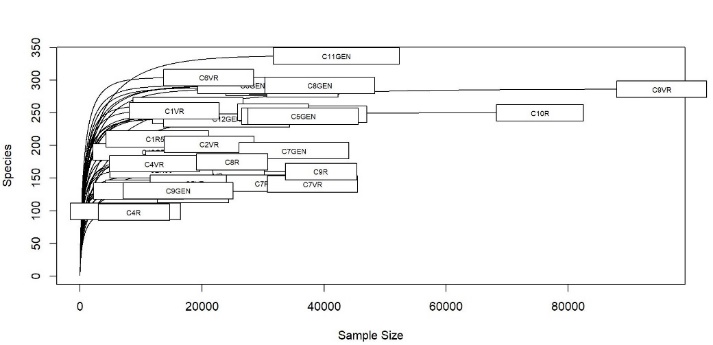


**Supplementary Tables**

**Supplementary Table 1.** metadata table used for the taxonomic analysis

| **Sample** | **Treatment** | **Site** | **SiteGroup** |
| --- | --- | --- | --- |
| C1GEN | Organic | Bulk | BulkO |
| C2GEN | Organic | Bulk | BulkO |
| C3GEN | Organic | Bulk | BulkO |
| C4GEN | Organic | Bulk | BulkO |
| C5GEN | Organic | Bulk | BulkO |
| C6GEN | Organic | Bulk | BulkO |
| C7GEN | Conventional | Bulk | BulkC |
| C8GEN | Conventional | Bulk | BulkC |
| C9GEN | Conventional | Bulk | BulkC |
| C10GEN | Conventional | Bulk | BulkC |
| C11GEN | Conventional | Bulk | BulkC |
| C12GEN | Conventional | Bulk | BulkC |
| C1VR | Organic | CloseRoot | CloseRootO |
| C2VR | Organic | CloseRoot | CloseRootO |
| C3VR | Organic | CloseRoot | CloseRootO |
| C4VR | Organic | CloseRoot | CloseRootO |
| C5VR | Organic | CloseRoot | CloseRootO |
| C6VR | Organic | CloseRoot | CloseRootO |
| C7VR | Conventional | CloseRoot | CloseRootC |
| C8VR | Conventional | CloseRoot | CloseRootC |
| C9VR | Conventional | CloseRoot | CloseRootC |
| C10VR | Conventional | CloseRoot | CloseRootC |
| C11VR | Conventional | CloseRoot | CloseRootC |
| C12VR | Conventional | CloseRoot | CloseRootC |
| C1R | Organic | Root | RootO |
| C2R | Organic | Root | RootO |
| C3R | Organic | Root | RootO |
| C4R | Organic | Root | RootO |
| C5R | Organic | Root | RootO |
| C6R | Organic | Root | RootO |
| C7R | Conventional | Root | RootC |
| C8R | Conventional | Root | RootC |
| C9R | Conventional | Root | RootC |
| C10R | Conventional | Root | RootC |
| C11R | Conventional | Root | RootC |
| C12R | Conventional | Root | RootC |

**Supplementary Table 2.** Permutational multivariant analysis of variance (PERMANOVA) based on Bray-Curtis dissimilarities of reads relative abundance of prokaryotic communities.

| **16S** | **Df** | **SS** | **R2** | **F** | **Pr(>F)** |
| --- | --- | --- | --- | --- | --- |
| **Treatment** | 1 | 0,7974 | 0,08827 | 3,4746 | 0,002 |
| **Site** | 2 | 0,7813 | 0,0865 | 1,7023 | 0,017 |
| **Treatment*Site** | 2 | 0,5697 | 0,06307 | 1,2412 | 0,138 |
| **Residual** | 30 | 6,8847 | 0,76216 |  |  |
| **Total** | 35 | 9,0332 | 1 |  |  |

**Supplementary Table 3. P**ermutational multivariant analysis of variance (PERMANOVA) based on Bray-Curtis dissimilarities of reads relative abundance of fungi communities.

| **ITS** | **Df** | **SS** | **R2** | **F** | **Pr(>F)** |
| --- | --- | --- | --- | --- | --- |
| **Treatment** | 1 | 1,1484 | 0,0903 | 3,3662 | 0,001 |
| **Site** | 2 | 0,6509 | 0,05118 | 0,954 | 0,596 |
| **Treatment*Site** | 2 | 0,6843 | 0,05381 | 1,003 | 0,432 |
| **Residual** | 30 | 10,2346 | 0,80472 |  |  |
| **Total** | 35 | 12,7182 | 1 |  |  |

**Supplementary Table 4.** Partitioning analysis conducted on environmental variables

|  | **16S** | | **ITS** | |
| --- | --- | --- | --- | --- |
|  | **R2** | **Adj R2** | **R2** | **Adj R2** |
| **Cu** | 0,07237 | 0,04509 | 0,05657 | 0,02882 |
| **pH** | 0,08275 | 0,05577 | 0,07717 | 0,05003 |
| **Na** | 0,07349 | 0,04624 | 0,04782 | 0,01982 |
| **Cu+pH** | 0,12477 | 0,07173 | 0,11302 | 0,05927 |
| **Cu+Na** | 0,11636 | 0,06281 | 0,09345 | 0,0385 |
| **pH+Na** | 0,11263 | 0,05885 | 0,12005 | 0,06672 |
| **All** | 0,1549 | 0,07567 | 0,15601 | 0,07688 |

**Supplementary Table 5.** dbRDA of bacterial and fungi population

|  | **16S** | | | **ITS** | | |
| --- | --- | --- | --- | --- | --- | --- |
|  | **SS** | **F** | **Pr(>F)** | **SS** | **F** | **Pr(>F)** |
| **Cu** | 0.788 | 3.3646 | 0.001 | 1.2285 | 3.7535 | 0.001 |
| **pH** | 0.4894 | 2.0899 | 0.016 | 0.6405 | 1.9569 | 0.001 |
| **Na** | 0.2614 | 1.1161 | 0.261 | 0.3758 | 1.1483 | 0.175 |
| **Residual** | 7.4944 |  |  | 10.4734 |  |  |

**Supplementary Table 6.** Comparison in the relative percentages of the prokaryotic phyla identified. Statistical analysis (T test) made on the number of the relative number of reads is reported (* *p*<0.05, ** *p*<0.01 *** *p*<0.001)

| Phylum | Organic | | Conventional | |  |  |
| --- | --- | --- | --- | --- | --- | --- |
|  | mean % | sd | mean % | sd | p-value |  |
| Proteobacteria | 20.88 | 3.12 | 25.57 | 5.09 | 0.0269 | * |
| Acidobacteriota | 28.68 | 6.32 | 23.11 | 5.19 | 0.03198 | * |
| Actinobacteriota | 10.38 | 3.59 | 12.24 | 4.70 | 0.1363 |  |
| Chloroflexi | 7.48 | 1.96 | 6.98 | 2.10 | 0.5926 |  |
| Bacteroidota | 4.66 | 1.13 | 6.30 | 2.62 | 0.03171 | * |
| Planctomycetota | 4.40 | 0.65 | 5.62 | 1.24 | 0.00453 | ** |
| Verrucomicrobiota | 6.30 | 2.08 | 5.16 | 1.76 | 0.06717 |  |
| Gemmatimonadota | 3.68 | 1.15 | 4.03 | 1.02 | 0.3326 |  |
| Myxococcota | 2.62 | 0.51 | 2.73 | 0.90 | 0.439 |  |
| Latescibacterota | 1.96 | 0.90 | 1.79 | 1.32 | 0.4102 |  |
| Methylomirabilota | 2.71 | 0.78 | 1.70 | 0.92 | 0.0006995 | *** |
| Patescibacteria | 0.47 | 0.33 | 0.81 | 0.56 | 0.03338 | * |
| NB1-j | 1.77 | 0.91 | 0.77 | 0.94 | 0.001324 | ** |
| RCP2-54 | 0.63 | 0.32 | 0.50 | 0.40 | 0.1381 |  |
| Armatimonadota | 0.19 | 0.13 | 0.47 | 0.23 | 0.09306 |  |
| Desulfobacterota | 0.76 | 0.50 | 0.47 | 0.27 | 0.02828 | ** |
| Firmicutes | 0.58 | 0.55 | 0.41 | 0.44 | 0.4157 |  |
| Nitrospirota | 0.88 | 0.39 | 0.40 | 0.16 | 0.06078 |  |
| Cyanobacteria | 0.38 | 0.65 | 0.34 | 0.56 | 0.9565 |  |
| Entotheonellaeota | 0.11 | 0.07 | 0.11 | 0.11 | 0.8621 |  |
| Bdellovibrionota | 0.07 | 0.12 | 0.09 | 0.05 | 0.8039 |  |
| MBNT15 | 0.19 | 0.17 | 0.09 | 0.09 | 0.01637 | * |
| Elusimicrobiota | 0.04 | 0.02 | 0.08 | 0.03 | 0.0006581 | *** |
| GAL15 | 0.07 | 0.07 | 0.07 | 0.08 | 0.8784 |  |
| Dependentiae | 0.05 | 0.04 | 0.05 | 0.04 | 0.9035 |  |
| Sumerlaeota | 0.01 | 0.02 | 0.03 | 0.03 | 0.006104 | ** |
| Fibrobacterota | 0.00 | 0.01 | 0.01 | 0.02 | 0.04099 | * |
| Zixibacteria | 0.01 | 0.01 | 0.01 | 0.02 | 0.1711 |  |
| Hydrogenedentes | 0.00 | 0.00 | 0.01 | 0.02 | 0.04886 | * |
| Dadabacteria | 0.01 | 0.01 | 0.01 | 0.02 | 0.7091 |  |
| WS2 | 0.00 | 0.00 | 0.01 | 0.01 | 0.04365 | * |
| SAR324_clade(Marine_group_B) | 0.01 | 0.01 | 0.01 | 0.01 | 0.6408 |  |
| Abditibacteriota | 0.00 | 0.00 | 0.00 | 0.01 | 0.1022 |  |
| FCPU426 | 0.00 | 0.01 | 0.00 | 0.00 | 0.593 |  |
| WPS-2 | 0.00 | 0.00 | 0.00 | 0.00 | 0.1068 |  |
| Halanaerobiaeota | 0.00 | 0.00 | 0.00 | 0.00 | 0.5366 |  |
| WS4 | 0.00 | 0.00 | 0.00 | 0.00 | 0.6162 |  |
| Deinococcota | 0.00 | 0.00 | 0.00 | 0.00 | 0.03712 | * |
| Spirochaetota | 0.00 | 0.00 | 0.00 | 0.00 | 0.1587 |  |
| Margulisbacteria | 0.00 | 0.00 | 0.00 | 0.00 | 1 |  |
| Fusobacteriota | 0.00 | 0.00 | 0.00 | 0.00 | 0.3244 |  |

**Supplementary Table 7.** Comparison in the relative percentages of the fungi phyla identified. Statistical analysis (T test) made on the number of the relative number of reads is reported (* *p*<0.05, ** *p*<0.01 *** *p*<0.001)

| Phylum | Organic | | Conventional | |  |  |
| --- | --- | --- | --- | --- | --- | --- |
|  | mean % | sd | mean % | sd | p-value |  |
| Ascomycota | 48.28 | 15.26 | 57.85 | 14.23 | 0.0306 | * |
| Basidiomycota | 28.03 | 12.74 | 20.07 | 15.71 | 0.6651 |  |
| Mortierellomycota | 14.81 | 14.76 | 15.37 | 8.85 | 0.3787 |  |
| Olpidiomycota | 3.50 | 14.71 | 0.02 | 0.06 | 0.3245 |  |
| Chytridiomycota | 2.43 | 2.84 | 2.07 | 3.36 | 0.82 |  |
| Rozellomycota | 1.03 | 1.01 | 0.91 | 1.32 | 0.6142 |  |
| Kickxellomycota | 0.66 | 0.49 | 0.66 | 0.65 | 0.2205 |  |
| Zoopagomycota | 0.37 | 0.45 | 0.27 | 0.39 | 0.4507 |  |
| Entomophthoromycota | 0.24 | 0.38 | 0.31 | 1.15 | 0.729 |  |
| Basidiobolomycota | 0.22 | 0.50 | 1.30 | 3.59 | 0.1384 |  |
| Mucoromycota | 0.16 | 0.19 | 0.09 | 0.09 | 0.677 |  |
| Entorrhizomycota | 0.09 | 0.13 | 0.02 | 0.03 | 0.01849 | * |
| Glomeromycota | 0.07 | 0.10 | 0.03 | 0.04 | 0.2283 |  |
| Blastocladiomycota | 0.07 | 0.23 | 1.00 | 2.45 | 0.08547 |  |
| Monoblepharomycota | 0.01 | 0.02 | 0.00 | 0.01 | 0.6319 |  |
| Aphelidiomycota | 0.01 | 0.02 | 0.00 | 0.00 | 0.1551 |  |
| Neocallimastigomycota | 0.00 | 0.00 | 0.01 | 0.03 | 0.3244 |  |
